# Prediction of habitat complexity using a trait-based approach on coral reefs in Guam

**DOI:** 10.1038/s41598-023-38138-1

**Published:** 2023-07-08

**Authors:** Sofia B. Ferreira, John H. R. Burns, Kailey H. Pascoe, Clifford A. Kapono, Andres J. Reyes, Atsuko Fukunaga

**Affiliations:** 1 MEGA Lab, College of Natural and Health Sciences, University of Hawaii at Hilo, Hilo, HI 96720 USA; 2 Center for Global Discovery and Conservation Science, Arizona State University, Hilo, HI 96720 USA; 3 Marine Scientist, NAVFAC Systems Command Marianas, Joint Region Marianas, Santa Rita, GU 96915 USA

**Keywords:** Marine biology, Community ecology

## Abstract

Scleractinian corals are primary contributors to the structural complexity of coral reef ecosystems. The structure derived from their carbonate skeletons underpins the biodiversity and myriad of ecosystem services provided by coral reefs. This study used a trait-based approach to provide new insights into the relationships between habitat complexity and coral morphology. Three-Dimensional (3D) photogrammetry techniques were used to survey 208 study plots on the island of Guam, from which structural complexity metrics were derived and physical traits of corals were quantified. Three traits at the individual colony level (e.g., morphology, size, and genera) and two site-level environmental characteristics (e.g., wave exposure and substratum-habitat type) were examined. Standard taxonomy-based metrics were also included at the reef-plot level (e.g., coral abundance, richness, and diversity). Different traits disproportionately contributed to 3D metrics of habitat complexity. Larger colonies with a columnar morphology have the highest contribution to surface complexity, slope, and vector ruggedness measure, whereas branching and encrusting columnar colonies have the highest contribution to planform and profile curvature. These results highlight the importance of considering colony morphology and size in addition to conventional taxonomic metrics for the understanding and monitoring reef structural complexity. The approach presented here provides a framework for studies in other locations to predict the trajectory of reefs under changing environmental conditions.

## Introduction

The structural complexity of an ecosystem provides the physical architecture to support high levels of biodiversity and availability of services. Scleractinian corals are the primary contributors to habitat complexity on coral reefs due to their ability to secrete structurally complex calcium carbonate skeletons^1^. Higher structural complexity in coral reefs has been linked to increased biodiversity, reef resilience, and coastal protection^2–4^. However, the increasingly rapid ecosystem degradation occurring throughout the global ocean is resulting in substantial losses in structural complexity and, consequently, biodiversity^5,6^. More structurally complex corals have shown to be more susceptible to disturbances including bleaching, crown-of-thorns starfish predation, and breakage or dislodgement from storms^7–9,28^. A holistic understanding of the key physical drivers of structural complexity is, therefore, of critical importance to understand and predict the trajectory of reefs under changing environmental conditions.

While the role of scleractinian (hard) coral in providing three-dimensional (3D) structure to the reef has received substantial attention^10–14^, the contribution of specific physical traits to habitat complexity has yet to be accurately measured. The two most common approaches to investigating the relationships among coral assemblages and habitat complexity utilize species composition or percent coral cover data^5,13,14^. However, such approaches inaccurately capture the morphological plasticity of scleractinian corals^15–17^, potentially missing important insights. Hard corals exhibit high intra- and inter-specific variation in morphologies, ranging from simple encrusting and dome-shaped colonies to complex (tree-like) branching and columnar colonies^15,18^. Such morphological differences can result in disproportionate contributions of certain coral morphologies to habitat complexity^5,14^, highlighting the importance of considering morphological features when studying reef complexity. Focusing solely on species composition or percent cover can be problematic as these metrics fail to capture the influence of morphological traits on overall habitat complexity. A trait-based approach is likely a more suitable method when examining the associations between coral assemblages and reef structural complexity.

Trait-based analyses have been broadly applied to plant communities^19,20^, terrestrial animals^21,22^, and pelagic ecosystems^23,24^, but less frequently to coastal ecosystems such as coral reefs^12,25^. This pattern is likely to change considering the recent publication of the first global coral trait database developed by Madin et al. (2016)^26^. A “trait” is any morphological, physiological, phenological, or behavioral characteristic of an organism^27^. Previous studies have applied a trait-based approach to better understand how different coral morphologies contribute to structural complexity^12,28,29^. However, these studies were conducted with a limited taxonomic and morphological scope, considering only a few coral genera or morphologies at a time, and potentially missing ecologically important insights. More work is needed to explore the generality of these findings across regions, particularly in ones with high coral diversity. Further, the lack of standardized methodologies used in previous studies for quantifying structural complexity or characterizing traits also hinders the ability to determine what constitutes a suitable set of traits for predicting structural complexity. Structure-from-motion (SfM), a form of photogrammetry, is a tool that has recently equipped scientists with the ability to create high-resolution and spatially accurate 3D reconstructions of marine habitats to aid in coral reef research^10,30–32^. SfM provides an accurate, cost-effective, and accessible tool for quantifying coral traits and structural complexity metrics in coral reefs. Beyond the 3D reconstruction, the photogrammetry workflow produces digital elevation models (DEMs, i.e., digital representation of a continuous surface with terrain elevation data) and orthomosaics (i.e., geometrically corrected mosaicked image), which allows for quantification of a large range of ecological and physical reefscape characteristics without the high computational cost of directly analyzing the 3D output^13,32–34^.

Here, we utilized DEMs and orthomosaics to evaluate whether structural complexity metrics derived from SfM 3D reconstructions from 208 reef plots in the West Pacific are capable of being modeled by coral colony traits representing morphology, size, and genera. Habitat physical characteristics such as wave exposure level and underlying substratum type were also accounted for, given that scleractinian corals exhibit high levels of morphological plasticity under different environmental conditions^18,35,36^. Traditional taxonomy-based metrics of coral abundance, genus richness, and genus diversity were also included for comparison. This study, leveraging SfM techniques, aims to identify a suite of colony traits and environmental characteristics to identify key drivers of habitat structural complexity on coral reefs. This study provides valuable insight into colony and reef traits that support high structural complexity, which in turn can be leveraged to predict how biodiversity and ecosystem services will be impacted by disturbance-induced loss of structurally complex corals.

## Methods

### Study site and 3D photogrammetry surveys

**Figure 1:**
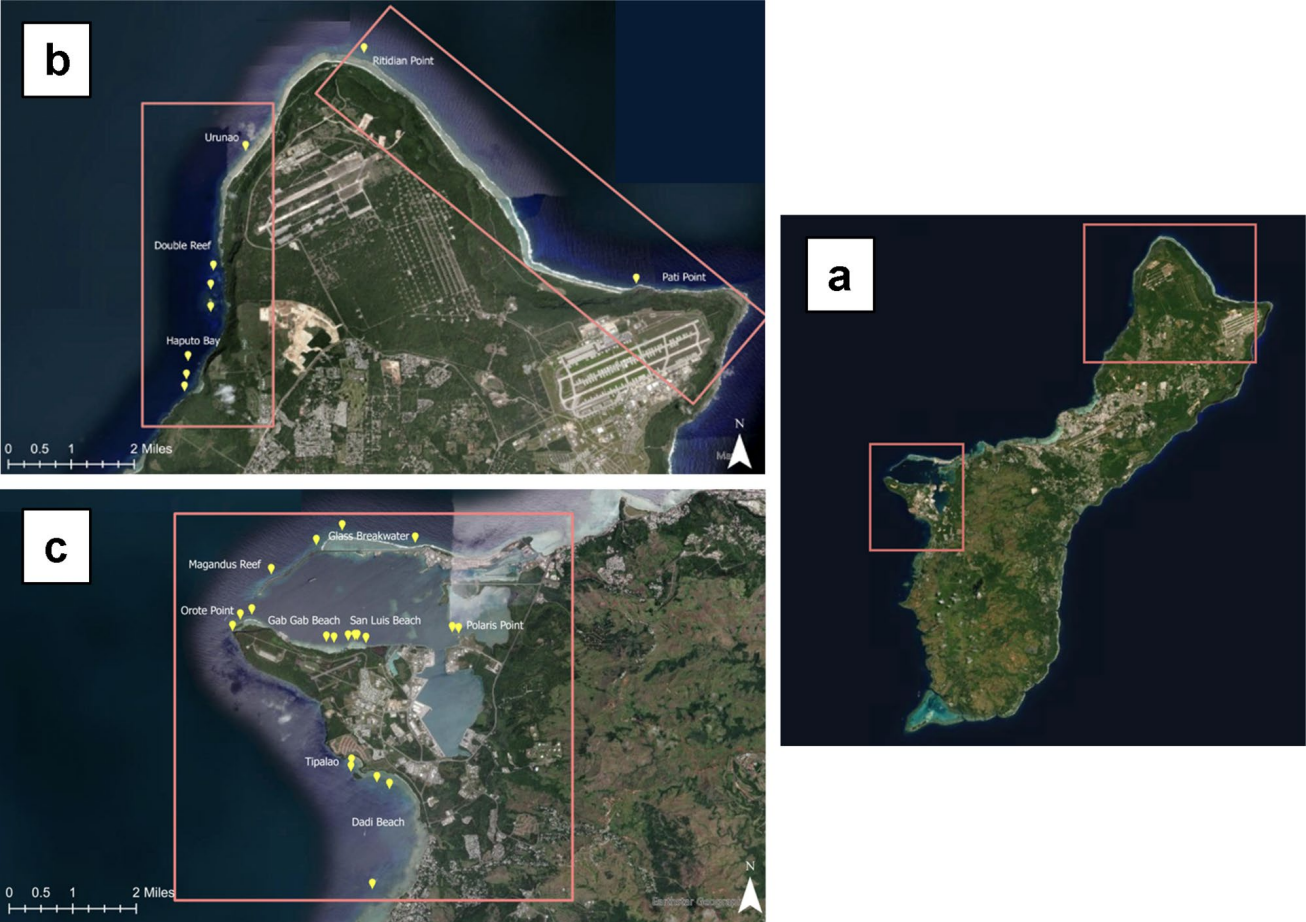
(**a**) Map of Guam island with study areas marked by a red box, (**b**) study sites (marked by yellow pins) located in the northwest and northeast coastlines, (**c**) study sites located in the west coast within and outside of Apra Harbor. Environmental Systems Research Institute (ESRI). ArcGIS Release 10.8. Redlands, CA. https://www.esri.com/ (2019).

The island of Guam is located in the Western Pacific at 13° 28′ N, 144° 45′ E. It is the southernmost island in the Mariana Archipelago, and the largest and most populated island in Micronesia. A total of 28 coral reef sites along Guam's west, northwest, and northeast coasts were surveyed in June of 2021 (Fig. 1). All sites were surveyed using Structure-from-Motion (SfM) photogrammetry techniques. Six to eight 2 × 2 m plots were surveyed at each study site by collecting continuous and overlapping (70–80%) images of the reef substratum via SCUBA and using a digital single lens reflex (SLR) camera at both planar and oblique angles. Scale bars with ground control points (GCPs) were placed at the corners of each plot for accurate scaling and orthorectification of the resulting 3D models. The 2 × 2 m plot size was used for these surveys as this work was part of a larger project characterizing coral communities on natural and artificial substrate provided by underwater cultural heritage sites^37^. The 4-m^2^ plots are capable of capturing coral assemblage metrics and metrics of 3D habitat complexity while enabling multiple sub-samples to be collected on specific substrate^38^. A total of 208 plots were surveyed. The camera used for the surveys was a Sony a7rlll with the ISO set to Auto.

The imagery was used to create 3D reconstructions of the reef plots using the software Agisoft Metashape v.1.7.1. (Agisoft LLC, St. Petersburg. Russia). Following the methodology of Burns et al. (2015)^7^, the process involved aligning the images and generating a sparse point cloud, building a dense point cloud, a polygon mesh model, overlaying a textured model, and finally rendering both a high-resolution two-dimensional (2D) orthomosaic projected from an overhead angle and a 2.5-dimensional Digital Elevation Model (DEM) at 1-cm raster resolution^10,33^. The Ground Sample Distance (GSD), the distance between two consecutive pixels which provides a measure of spatial resolution for 3D photogrammetric reconstructions, ranged from 0.186 to 0.418 mm/pixel on the orthorectified DEMs and orthomosaics produced in this study. Each orthorectified mosaic was layered with the DEM for further analyses of habitat community composition and habitat structural complexity.

### Characterization of coral and site traits

**Figure 2:**
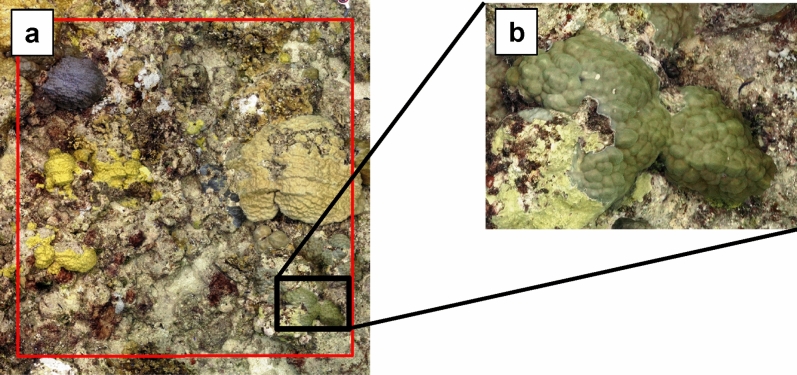
(**a**) Example of 1-cm raster resolution 2D orthomosaic with a 2 × 2-m plot overlaid in ArcMap. (**b**) Example of individual coral colony observed within the plot.

The two-dimensional (2D) orthomosaics and DEMs were imported into the geospatial software ArcMap v.10.8 (ArcGIS 10.8, Environmental Systems Resource Institute, Redlands, USA) to characterize coral traits. A 2 × 2 m polygon was overlaid over each orthomosaic using the create features tool in ArcMap to ensure that all analyzed plots are the exact same size (Fig. 2a). Every coral colony within the 2 × 2 m plot was identified down to genus level (Fig. 2b). The ahermatypic (non-reef-building) coral *Sinularia* and zoanthid *Palythoa* were also included due to their regular presence. Additional information recorded for each colony were morphology and size. The colony size was measured in centimeters (cm) using the ruler tool by measuring the longest diameter of each coral colony, from which a size category was determined. Size categories were 0–5 cm, 6–10 cm, 11–20 cm, 21–30 cm, 31–40 cm, 41–50 cm, 51–60 cm, 61–70 cm, 71–80 cm, 81–90 cm, 91–100 cm, and > 1 m. This sizing method was used due to its practicality and applicability to many in-situ coral reef surveys where the longest diameter of each coral colony is visually estimated or measured by a ruler to determine colony sizes^39,40^. Moreover, the utilization of alternative approaches such as manually delineating colonies to measure colony areas would have resulted in a considerable expenditure of time and proved impractical in the context of large-scale surveys. Recorded morphologies included mounding, encrusting-flat, encrusting-columnar, laminar, laminar-columnar, columnar, knobby, branching, bifacial, mounding-lobate, and free-living based on the classification by Winston et al. (2018)^38,39^.

Environmental characteristics were noted for each of the 28 reef sites and included wave exposure and substratum type. Wave exposure was categorized as either Low, Moderate, or High and was based on a combination of wind and current patterns around the island, as well as physical characteristics observed at each survey site. This classification system was supported by expert knowledge and corroborated by existing literature. For instance, sites classified as having “High” wave exposure are subject to strong waves generated by the east-facing trade winds, which persistently impact the northern and eastern coasts of Guam year-round^41,42^. Sites categorized as having “Moderate” wave exposure face westward, sheltered by the lee side of trade wind forcing and thus resulting in generally lower wind speeds and smaller wave heights^43^. Finally, sites classified as having “Low” wave exposure are facing westward or southward shores and are further protected by physical barriers such as a break wall or enclosed embayment within and around Apra Harbor^43^. Substratum types were assigned based on categories described in Kendall and Poti^44^. Out of the seven possible categories, three were identified in the present study: aggregate reef, pavement, and rock/boulder.

### Quantification of structural complexity

All DEMs were cropped using custom scripts written in R with the raster and sf packages^45–47^. The 2 × 2-m digitized polygons used in ArcMap to demarcate each survey plot were exported as shapefiles and imported into R to define the extent to which the DEMs were cropped. This ensured the 3D metrics extracted from each plot were accurately collocated with the spatial area used to characterize the coral traits. Using custom R scripts and the procedure of Fukunaga et al.^33^, the following structural complexity metrics were calculated from the cropped DEMs for each survey plot: surface complexity, slope, vector ruggedness measure (VRM), profile curvature, and planform curvature. These metrics are computed using 3 × 3 cell windows of the DEM raster throughout the entire area of the DEM to produce a single average value or distribution of values for each 2 × 2-m study plot.

### Statistical analyses

Statistical analyses were completed in the statistical software R v.4.1.1 (R core team 2019) running in RStudio v.1.4.1717-3^45^. All categorical traits (e.g., size, morphology, and genus) data were summarized by summing up the total count of coral colonies within each size, morphology, or genus category per plot. Coral abundance (i.e., the number of individual colonies), coral genus richness and diversity (Shannon Index H’) were also calculated for each reef plot. These data were then scaled and centered using the scale function in R.

A series of general linear models were built for each structural complexity metric to investigate relationships between coral traits, environmental characteristics, and the metrics of habitat complexity. A multiple regression model with stepwise selection by Akaike Information Criterion (AIC) was encoded for each complexity metric with each of the three trait groups (e.g., genus, size, and morphology). The coral assemblage metrics (e.g., coral abundance, richness, and diversity) exhibited high collinearity, thus each of these metrics was used to run a simple linear regression model with each complexity metric. For the environmental parameters, structural complexity metrics were first log-transformed and averaged for each site, and the analysis of variance (ANOVA) tests were performed to test for differences among either wave exposure levels or substratum types. Lastly, the Multivariate Redundancy Analysis (RDA) using the vegan package was performed to determine the relationships between all coral traits and all structural complexity metrics^48^. Due to multicollinearity among predictor variables, a stepwise forward selection with 999 permutations was performed to select the most robust RDA model. A permutation test was then used to test for the significance of the resulting RDA model and each individual variable.

## Results

The 208 plots resulted in 12,897 individual coral colonies that were digitally annotated and surveyed. A total of 32 coral genera and 11 morphotypes were recorded. The number of coral colonies per 2 × 2 m plot ranged from 1 to 233, with an average of 62 colonies per plot. The five most common coral genera observed were *Porites*, *Leptastrea*, *Astreopora*, *Goniastrea,* and *Dipsastrea* (Fig S1). The five most common morphotypes observed were Mounding, Encrusting Flat, Laminar Columnar, Branching, and Encrusting Columnar (Fig S2).

**Figure 3:**
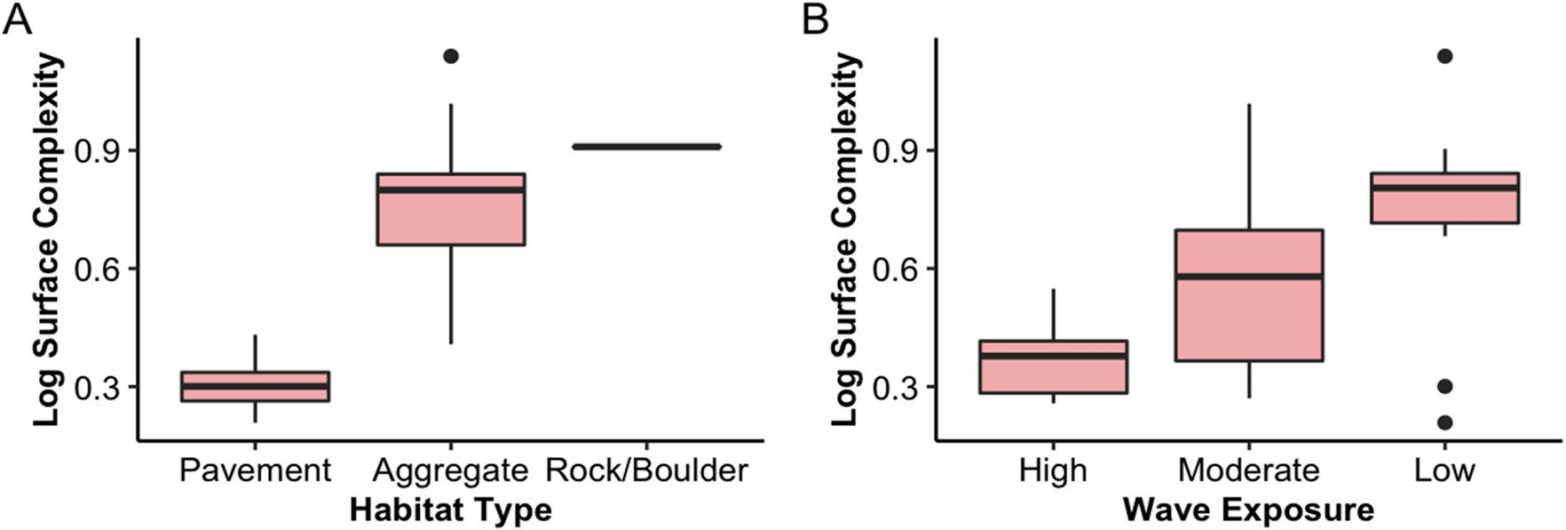
Box plots showing (**A**) Log-transformed Surface Complexity by substratum type (n = 28, ANOVA p < 0.001), and (**B**) Log-transformed Surface Complexity by wave exposure type (n = 28, ANOVA p < 0.001).

Coral genus diversity (H’), genus richness, and coral abundance each showed a significant negative relationship with surface complexity, VRM, and slope (Table 1). Profile and planform curvature, however, showed a significant positive relationship with genus diversity and richness, and mean curvature showed no significant relationship with any coral assemblage metric (Table 1). The multiple linear regression models showed that, of the 32 coral genera, *Pocillopora* and *Millepora* were the only genera to exhibit a positive relationship with a complexity metric: *Pocillopora* with the curvature metrics and *Millepora* with surface complexity and slope (Table 2). Additionally, seven genera exhibited a significant negative relationship with one or more of the complexity metrics of surface complexity, VRM, and slope (Table 2). For colony size, statistically significant positive associations were identified between complexity metrics and seven out of the ten size classes, with the exception of the 0–5 cm size class, which demonstrated a negative association with complexity metrics (Table 2). Similarly, most morphology classes showed significant associations with various complexity metrics (Table 2). The ANOVA tests revealed that surface complexity significantly differs among both substratum types and wave exposure levels, indicating higher complexity at Aggregate, and Rock and Boulders sites as well as sites with lower wave energy (Fig. 3A and 3B).

**Table 1:** Results of simple linear regression for each structural metric with each of the coral assemblage metrics. Plus ( +) sign indicates positive relationship, and minus (−) sign indicates negative relationship. “ns” means not significant. *, ** and *** denote p-values that are less than 0.05, 0.01 and 0.001, respectively. Surf. Complexity; Surface Complexity, Plan. Curvature; Planform Curvature, Prof. Curvature; Profile Curvature.

Coral Assemblage Metric	Surf. Complexity	VRM	Slope	Prof. Curvature	Plan. Curvature
Genus Diversity (H’)	−***	−***	−***	+**	+**
R^2^	0.33	0.44	0.33	0.04	0.04
Genus Richness	−***	−***	−***	+*	+*
R^2^	0.26	0.35	0.22	0.02	0.02
Coral Abundance	−***	−***	−***	ns	ns
R^2^	0.11	0.21	0.09

**Table 2:** Results from the multiple linear regressions for (A) Size, (B) Morphology, and (C) Genera. Plus ( +) sign indicates positive relationship, and minus (−) sign indicates negative relationship. Empty cells indicate that the variable was not significant. *, ** and *** denote p-values that are less than 0.05, 0.01 and 0.001, respectively. Explanatory variables that did not show a statistically significant relationship with any of the complexity metrics are not included in the table. Surf. Complexity; Surface Complexity, Plan. Curvature; Planform Curvature, Prof. Curvature; Profile Curvature.

Trait	Surf. Complexity	VRM	Slope	Prof. Curvature	Plan. Curvature
Size					
Adj-R^2^	0.52	0.58	0.50	0.09	0.09
	0–5 cm	−**	−**	−***	
	6–10 cm				
	11–20 cm	+**		+***	
	31–40 cm				
	41–50 cm	+*		+***	+***
	51–60 cm	+**	+***	+**	
	61–70 cm	+*	+**	+*	
	71–80 cm		+*		
	81–90 cm			+*	
	> 1 m	+***	+***	+***	
Morphology					
Adj-R^2^	0.34	0.39	0.32	0.05	0.06
	Branching			+**	+**
	Encrusting Columnar		+**	−**	−**
	Laminar			+*	
	Columnar	+*	+**	+**	
	Laminar Columnar	+**		+**	
	Encrusting Flat	−***	−***	−***	
	Mounding	−***	−***	−***	
Genera					
Adj-R^2^	0.32	0.36	0.42	0.06	0.07
	*Astrea*	−*			
	*Astreopora*	−***	−***	−***	
	*Cyphastrea*	−*	−***	−**	
	*Leptastrea*	−***	−***	−***	
	*Pocillopora*		−*	+**	+**
	*Montipora*		−*	−***	
	*Millepora*	+*	+*	−*	−*
	*Sinularia*	−***	−**	−***

**Figure 4:**
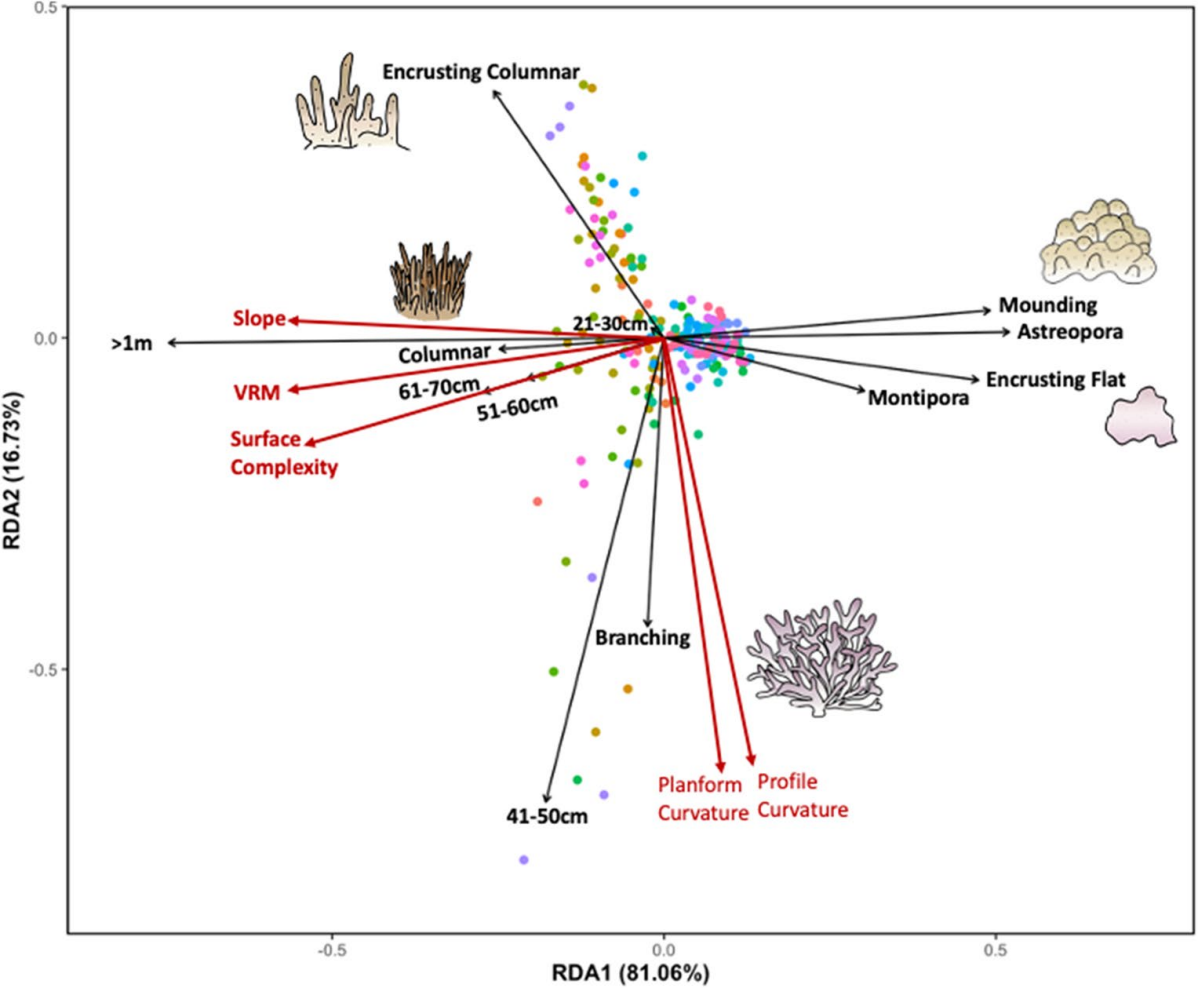
Redundancy Analysis (RDA) biplot with type 2 scaling showing the relationships among structural complexity metrics (red vectors) and coral traits (black vectors). The dots represent each of the individual study plots (n = 208) and are colored by site. The first two axes explained 81.06% and 16.73% of the explained variation within the data, respectively (Adj R^2^ = 0.45, Permutest p = 0.001). A list of each parameter’s variance explained, effect size, and significance level is given in Table 3.

After completing the forward stepwise model selection with 999 permutations the final Redundancy Analysis (RDA) included 12 predictor variables; 5 size categories, 5 morphologies, and 2 genera (Table 3). The optimal RDA resulted in the first two axes capturing 81.06% and 16.73% of the explained variation within the data, respectively (Adj R^2^ = 0.45, Permutest p = 0.001). Slope, VRM, and Surface Complexity showed a strong positive association with the size class categories of 51-60 cm, 61-70 cm, and greater than a 1 m, and with the Columnar morphology (Fig. 4, Table 3). Conversely, these complexity metrics exhibited a negative association with the genera *Astreopora* and *Montipora*, and the morphologies of Mounding and Encrusting-Flat (Fig. 4, Table 3). Planform and Profile curvature exhibited a positive association with the Branching morphology and the 41-50 cm size class, as well as a negative association with the Encrusting-Columnar morphology (Fig. 4, Table 3). These relationships closely matched those identified in the univariate analyses (Table 2).

**Table 3:** Summary table of statistical results of the redundancy analysis (RDA). Asterisks denote significance level with *, ** and *** signifying p-values that are less than 0.05, 0.01 and 0.001, respectively.

Variable	Variance explained	F	Significance level
> 1 m	1.09624	82.5582	***
41-50 cm	0.30160	22.7131	***
51-60 cm	0.09834	7.4058	***
61-70 cm	0.06119	4.6083	**
21-30 cm	0.06110	4.6011	**
Encrusting Columnar	0.33400	25.1534	***
Encrusting Flat	0.06750	5.0836	**
Branching	0.10408	7.8382	***
Mounding	0.04105	3.0911	*
Columnar	0.04472	3.3676	*
Astreopora	0.13914	10.4783	***
Montipora	0.06176	4.6514	*

## Discussion

This study found that various coral traits disproportionately contribute to three-dimensional structural complexity on coral reefs in Guam. Understanding the coral traits that predict complexity in reefs can help forecast how reef structure and associated ecological functions will be impacted by changes in coral assemblage. Coral colony size largely influenced the structural metrics of Surface Complexity, Vector Ruggedness Measure (VRM), and Slope (Table 2). Increases in the number of larger coral colonies yielded higher structural complexity values, whereas increases in the number of small colonies (e.g., 0—5 cm in size) resulted in lower structural complexity. Similarly, a comprehensive study among Seychelles, Maldives, Chagos, and the Great Barrier Reef showed that maximum colony size was a positive predictor of reef structural complexity^12^. Notably, our study in Guam produced new insight that coral colony size needs to be greater than 10 cm, based on our univariate analysis of the size trait (Table 2), or even larger, based on our multivariate analyses collectively considering all traits, for increases in their abundance to contribute to reef structural complexity positively in Guam (Table 3). This has an important implication in conservation as a large number of coral recruits are unlikely to contribute to structural complexity in the same way as a few large colonies do, despite having similar total live coral cover. This result also highlights the inability of 2D ‘total live coral cover’ to capture nuances in how live coral positively influences structural habitat complexity, and thus caution should be used when solely relying on this metric to assess reef condition.

Aside from providing greater structural complexity, larger colonies are also generally more resistant to diseases, dislodgement, and mortality^29,49^, highlighting the importance of large corals not only for structural complexity but also for reef resilience and function^50–52^. As a result of environmental change and anthropogenic stress, coral reef ecosystems in the Great Barrier Reef, for example, are already showing a shift towards the dominance of smaller coral colonies^53^. If such an event occurs in Guam or elsewhere, it could result in a significant loss of structural complexity and thus hinder associated ecosystem services. It is important to note that we measured size as the length of an entire colony measured across the maximum diameter, which is a conventional metric of colony size^39,40^. While ecologically useful, this metric does not capture the true 2D or 3D size of a colony, thus future studies can utilize full semantic segmentation of corals to more accurately capture how colony size, as a trait, influences reef habitat complexity.

Coral morphology is a key driver of many biological and ecological processes in reef ecosystems^12,51,52,54^. In the present study, Columnar, Encrusting-Columnar, Laminar-Columnar, and Branching were the coral morphologies that exhibited the highest positive contribution to structural complexity (Tables 2, 3). Columnar, Encrusting-Columnar, and Laminar-Columnar were predominantly observed in *Porites* corals, whereas Branching was observed in *Pocillopora* corals, matching previous studies in Guam reefs^55^ (Fig S8). Increases in the number of colonies with either Mounding or Encrusting-Flat morphology, on the other hand, showed strong negative effects on structural complexity (Tables 2, 3). This finding contradicts a previous study in the Northwestern Hawaiian Islands (NWHI) where positive correlations between the abundances of these morphologies and structural metrics were found^56^. A possible explanation for this is that in contrast to this study that accounted for twelve morphological types, the study in the NWHI only had four, branching, tabulate, mounding, and encrusting morphologies, as morphologies such as columnar and laminar columnar are rare in the NWHI. This could result in differences in the overall ranges of individual complexity metrics between the two studies, potentially affecting the results of analyses. Another potential explanation is the dominance of small colonies and overall lack of large colonies in the mounding and encrusting-flat morphologies in the present study (Fig S4). Further investigations are required among various regions and reef types to unravel the effects of coral morphology and size on reef complexity dynamics.

Profile and Planform Curvature exhibited a positive relationship with the abundance of Branching corals (Table 2, Fig. 4). These findings are also contrasted by another previous study in the NHWI where Branching corals were positively correlated with Surface Complexity and Slope^13^. In addition to the differences in the morphological types as discussed above, such disparate results can also be explained by methodological differences pertaining to study plot sizes. The plot size in the study in the NWHI was 90 m^2^, whereas the size in the present study was 4 m^2^. Averaging complexity metrics over 90 m^2^ likely results in accounting for more components on the reefs compared to averaging over 4 m^2^ simply due to the larger spatial coverage. As curvature values can sometimes take extreme values^57^, plot sizes can affect curvature metrics more than other complexity metrics (e.g., slope and VRM) when they are averaged for a plot. In another study, Darling et al. found that proportions of Branching corals on reefs were negatively correlated with reef complexity scores based on visual estimation^12^. As coral survey methodologies can vary among different studies (e.g., classifications of coral morphology, plot sizes, and calculation/estimation of complexity metrics), generalizing findings from different studies may be difficult, highlighting the importance of being explicit about the survey methodologies when reporting the associations between coral and structural complexity. The variability among these findings highlights the importance of conducting a thorough analysis of coral assemblage composition and complexity for any site or region, as there is unlikely to be a singular metric or assumption that can be applied to any location to understand how live corals influence habitat structure.

**Figure 5:**
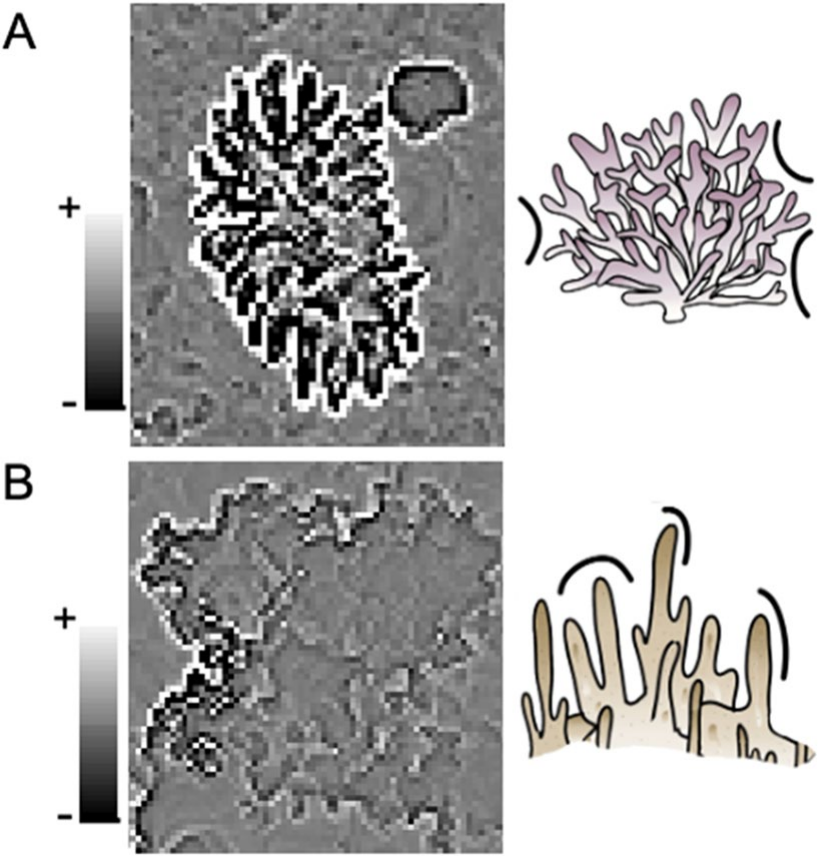
Top-Down view example of profile curvature rasters for (**A**) Branching and (**B**) Encrusting Columnar showing the convex vs concave structures, respectively (i.e., the structural patterns found in the univariate and multivariate analyses).

Aside from being positively correlated with Branching morphology, Profile and Planform curvature also showed a negative relationship with Encrusting-Columnar morphology (Table 2). Curvature is a non-monotonic measure, with “zero” meaning a flat surface, whereas values less and greater than zero indicating increases in complexity; negative values indicate a convex surface and positive values indicate concave surface^58^. Our results thus suggest that an Encrusting-Columnar morphology in the present study had more convex surfaces, and Branching morphology had more concave surfaces. This is supported by visualization of Profile Curvature for the two morphology types where convex surfaces around Branching morphology yielded extreme positive values (Fig. 5A) and Encrusting Columnar yielded moderate to negative values (Fig. 5B). The ability of curvature metrics to separate these two seemingly similar morphology types is a key finding that has not been previously documented due to the lack of Encrusting-Columnar morphology in the previous studies at other geographic locations utilizing photogrammetry techniques^13,56^.

Despite the high coral biodiversity of coral reefs surrounding the Island of Guam, coral abundance, genus diversity and richness had significant inverse relationships with Surface Complexity, VRM, and Slope (Table 1). Profile and Planform Curvature, on the other hand, did show a weak yet positive relationship with coral richness and diversity (Table 1). When looking at the contribution of the dominant genera in our study, however, the dominance of a particular genera also did not seem to explain changes in complexity. This contradicts a study in the Caribbean, for example, which found that the dominance of one or two coral genera showed the highest contribution to reef structural complexity compared to more diverse sites^5^. For instance, while the genus Porites accounted for 49.6% of the coral colonies surveyed for this study, Porites alone did not exhibit a positive relationship with any complexity metric. As Porites corals exhibited nine out of the twelve possible morphologies, it is possible that the variability in Porites morphologies confounded the effect of this genus on structural complexity when analyzed collectively (Fig. S8). It is also important to note that coral abundance in the present study was measured by colony density per 4-m^2^ plot. Having more colonies, and possibly more species, in each plot can lead to individual colonies in the plot being smaller in size, which has negative effects on structural complexity (Table 2).

Only two of the 32 surveyed genera showed a positive relationship with a complexity metric: *Pocillopora* with Profile and Planform Curvature, and *Millepora* with Surface Complexity and Slope (Table 2). In the case of *Pocillopora*, this relationship could be linked to morphological traits, as *Pocillopora*’s dominant morphology is Branching, which also exhibited a positive relationship with Profile and Planform Curvature (Table 2, Figs. S8 and S9). This is consistent with a recent study in Oahu, Hawaii where the associations between structural complexity and specific coral species were found to be closely tied to species morphology^59^. *Millepora*, on the other hand, exhibited some variability in both sizes and morphologies, but they lacked small colonies (i.e., 0–5 cm in size, Fig S5). This might have contributed to the observed positive associations with Surface Complexity and Slope, indicating the importance of considering colony size when modeling habitat complexity on coral reefs. Additionally, seven coral genera exhibited strong negative relationships with complexity metrics (Table 2). These patterns may also be linked to morphological coral traits, as these genera exhibited primarily encrusting flat or mounding morphologies and small colony sizes (Figs S8 and S9). Collectively, our results overall indicate that coral genera alone are not a strong predictor of habitat complexity in Guam’s reefs. These findings highlight the increased importance of accounting for coral morphological traits as it suggests that taxonomy-based metrics alone does not provide sufficient insights to capture the high morphological plasticity of hard corals and its effects on reef complexity, as observed in this study. Future studies should focus on examining interactions between specific morphology types and their sizes in the effects on reef structural complexity and consider species data in relation to morphological traits.

Environmental parameters also influenced structural complexity in Guam reefs, as we observed sites with higher wave exposure to have significantly lower structural complexity values (Fig. 3B). This could be explained by the wave-forcing, resulting in shifts in the abundance and size of various morphologies. Hard corals exhibit strong morphological plasticity in response to hydrodynamic force^15,18,50^. This occurs because certain morphologies, such as those that grow vertically and have a smaller attachment, have a higher risk of dislodgement and mechanical damage in higher wave energy environments^49^. For our study sites in Guam, the sites with the highest wave energy tended to be dominated by smaller coral colonies with a mounding morphology, whereas the sites with less wave energy were dominated by larger colonies with columnar and laminar morphologies (Figs S6 & S7). Lastly, as expected, structural complexity was much lower at pavement-type reefs than aggregate and rock and boulder reefs (Fig. 3A), highlighting the role that underlying substrate can play in the reef’s architectural complexity^57^.

Coastal ecosystems are presently among the most heavily impacted ecosystems on earth^60^. Natural and anthropogenic disturbances are rapidly reconfiguring coral assemblage composition, urging the need to identify key physical drivers of habitat complexity given their direct link to ecosystem functionality^2^. Using a trait-based approach, we examined how physical coral traits influence 3D photogrammetry-derived structural complexity metrics and compared the results with those based on either taxonomic classifications or abundance/diversity metrics. Our main conclusion is that the traits of coral colony size and morphology are strong predictors of habitat complexity in Guam’s reefs and should thus be included in coral reef monitoring programs. This study offers important insights and foundation for future studies assessing the impact of changing reef habitats on reef-associated organisms under climate change.

## Supplementary Information

Supplementary Information.

## Data Availability

The datasets generated and analyzed during the current study are available from the corresponding author on reasonable request and permission of NAVFAC.
