# Supplementary material for: Prediction of habitat complexity using a trait-based approach on coral reefs in Guam
Source: Sci Rep. 2023 Jul 8;13:11095. doi: 10.1038/s41598-023-38138-1 (PMC10329656; doi:10.1038/s41598-023-38138-1)
Supplement: Supplementary file 1 — Supplementary Information. [file 41598_2023_38138_MOESM1_ESM.docx]

**Prediction of habitat complexity using a trait-based approach on coral reefs in Guam**

**Authors:** Sofia B. Ferreira^1, *^, John H.R. Burns^1^, Kailey H. Pascoe^1,2^, Clifford A. Kapono^1,2^, Andres J. Reyes^3^, Atsuko Fukunaga^1,2^.

**Affiliations:**

1. MEGA Lab, College of Natural and Health Sciences, University of Hawaii at Hilo, Hilo HI 96720, USA.
2. Center for Global Discovery and Conservation Science, Arizona State University, Hilo HI 96720, USA.
3. Marine Scientist, NAVFAC Systems Command Marianas, Joint Region Marianas, Santa Rita, GU 96915, USA. (ALTHOUGH THE MATTER HEREIN MAY DEAL WITH ONGOING OR ANNOUNCED POLICIES AND PROGRAMS OF THE NAVY, CONTRIBUTIONS BY THIS COAUTHOR ARE THE AUTHOR’S PERSONAL VIEWS AND NOT NECESSARILY THOSE OF THE DEPARTMENT OF DEFENSE OR ITS COMPONENTS.)

* Correspondence: sofiaf@hawaii.edu; Tel.: +1-808-217-6482

**Supplementary Information**

**
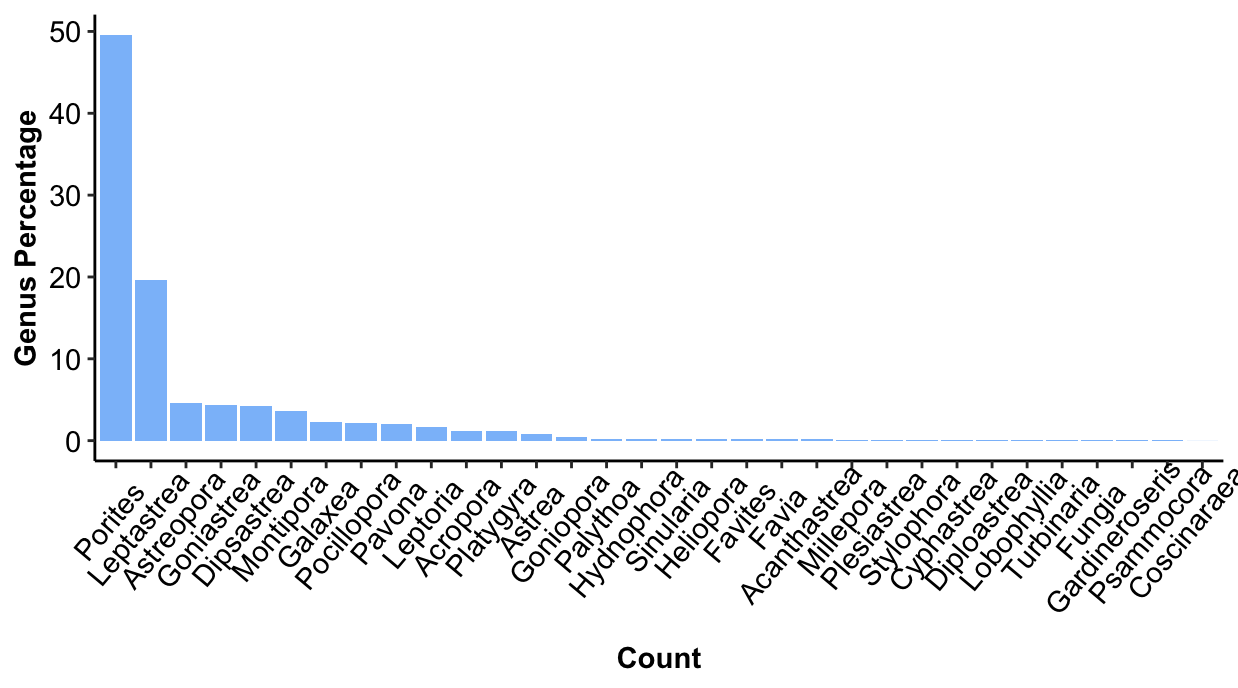
**

**Supplementary Figure 1.** Total proportion of each genus based on all 12,897 coral colonies surveyed.


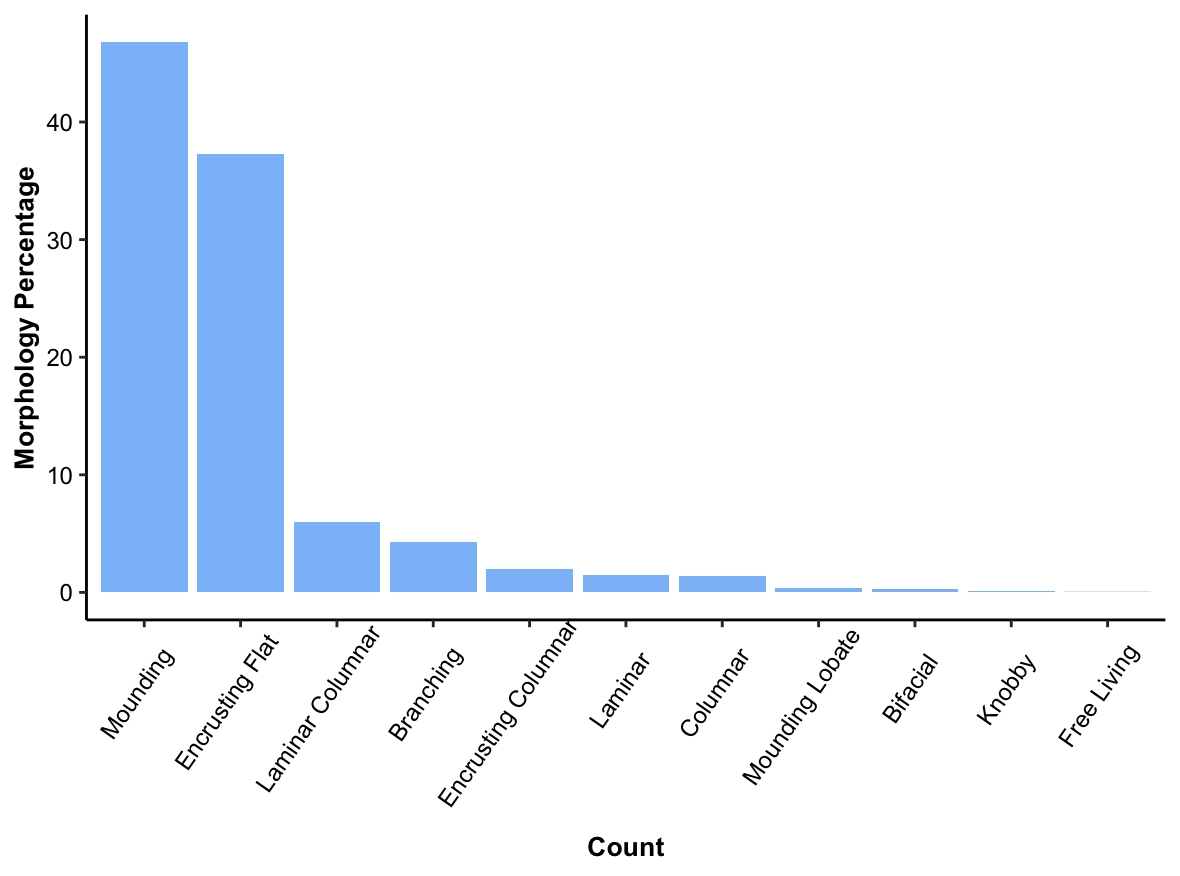
**Supplementary Figure 2.** Total proportion of each morphology based on all 12,897 coral colonies surveyed.

**
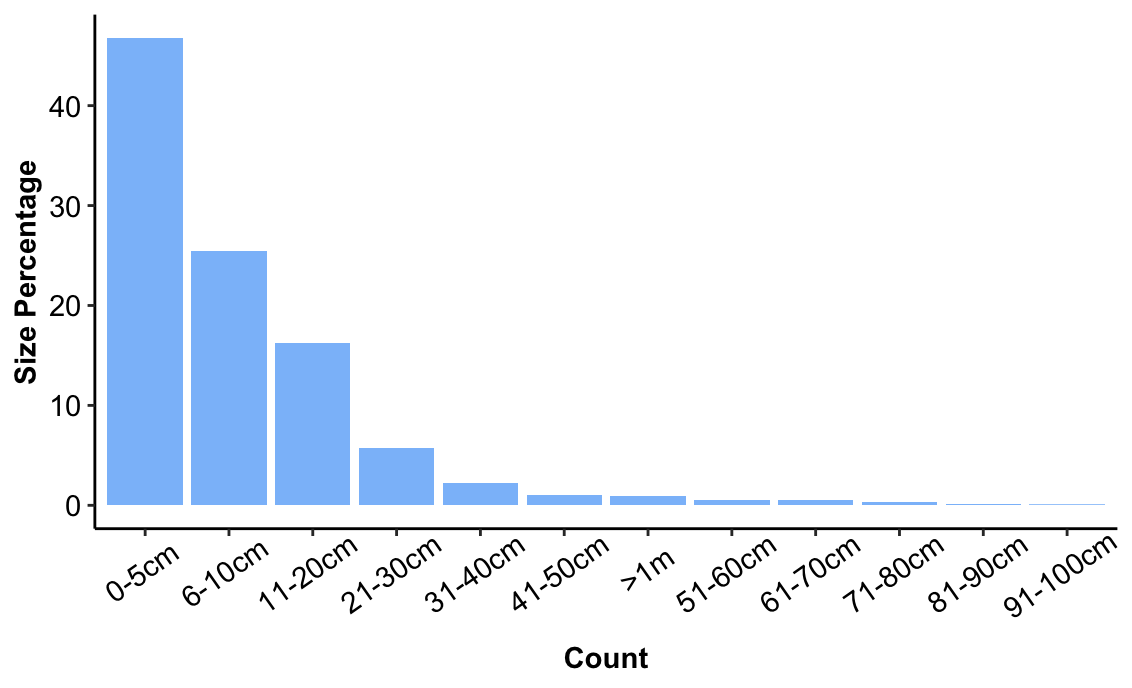
**

**Supplementary Figure 3.** Total proportion of each size category based on all 12,897 coral colonies surveyed.


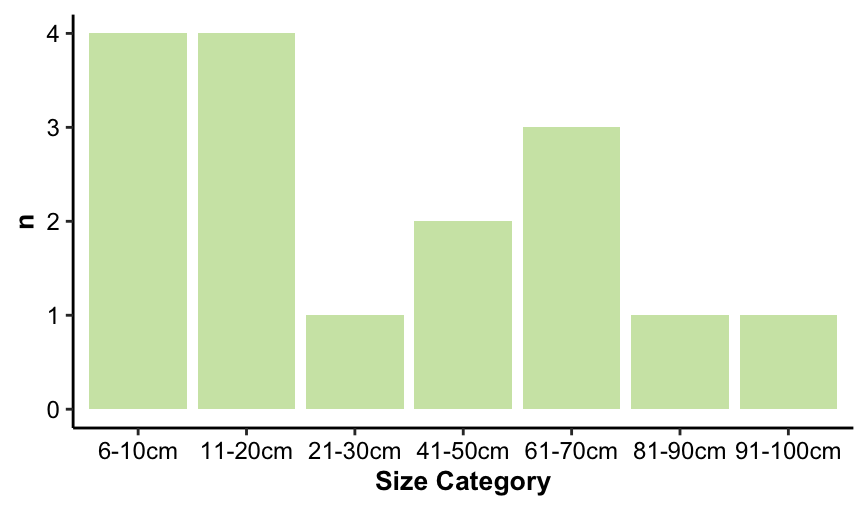

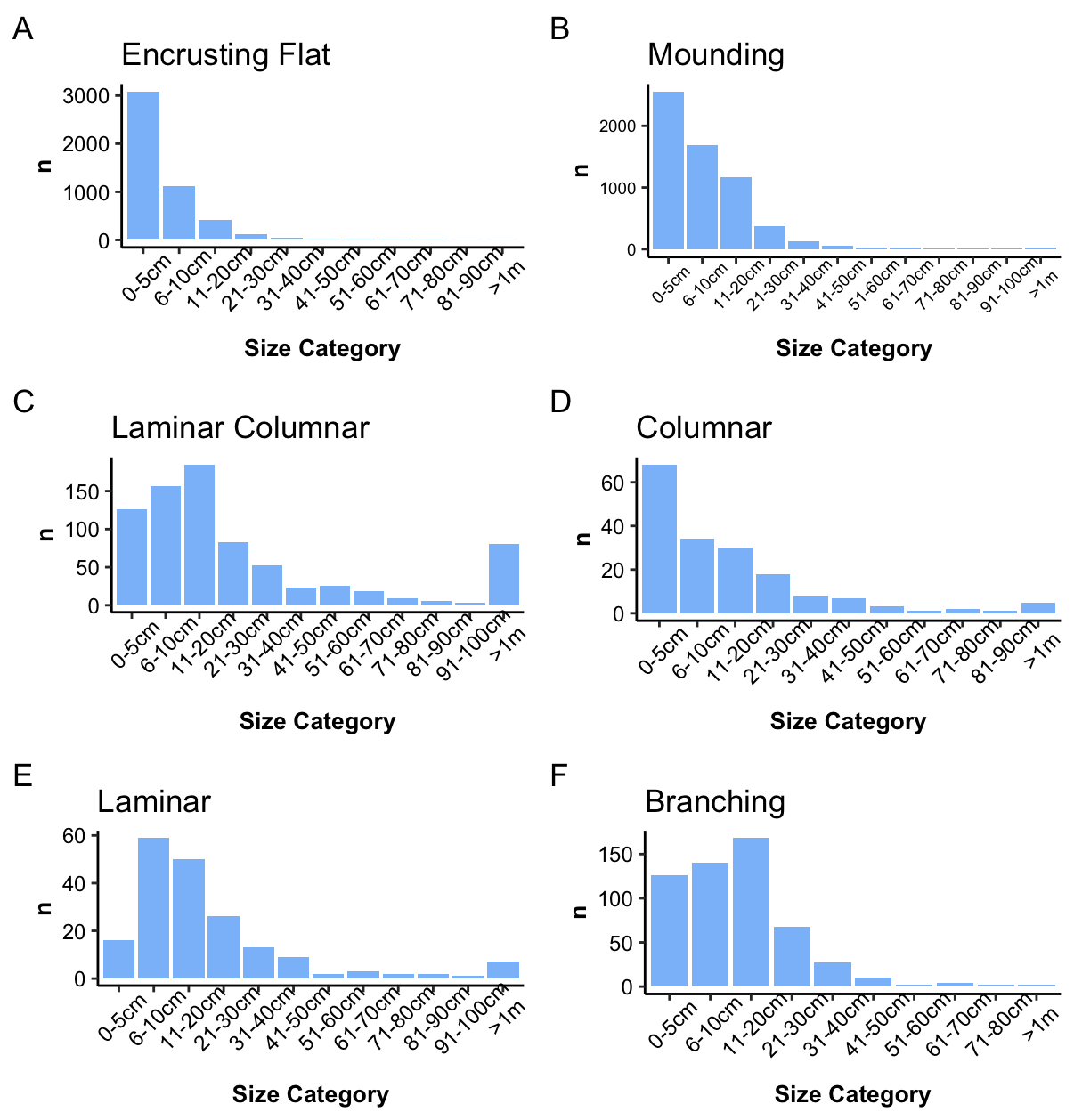
**Supplementary Figure 4.** Size class distribution for (A) Encrusting Flat, (B) Mounding, (C) Laminar Columnar, (D) Columnar, (E) Laminar, and (F) Branching.


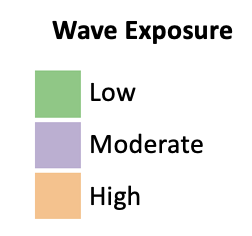
**Supplementary Figure 5.** Size class distribution for *Millepora* corals.


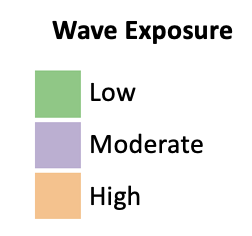

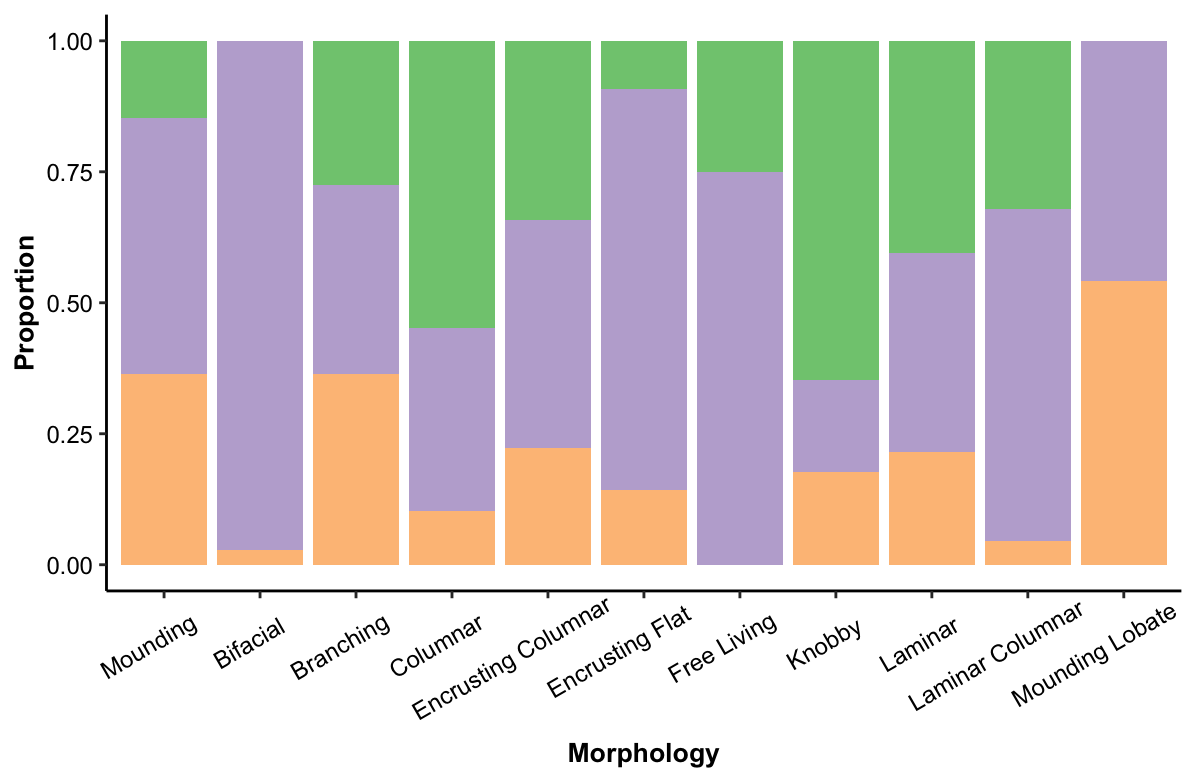
**Supplementary Figure 6.** Stacked bar chart showing proportion of each morphology category based on wave exposure levels.


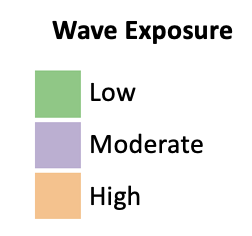

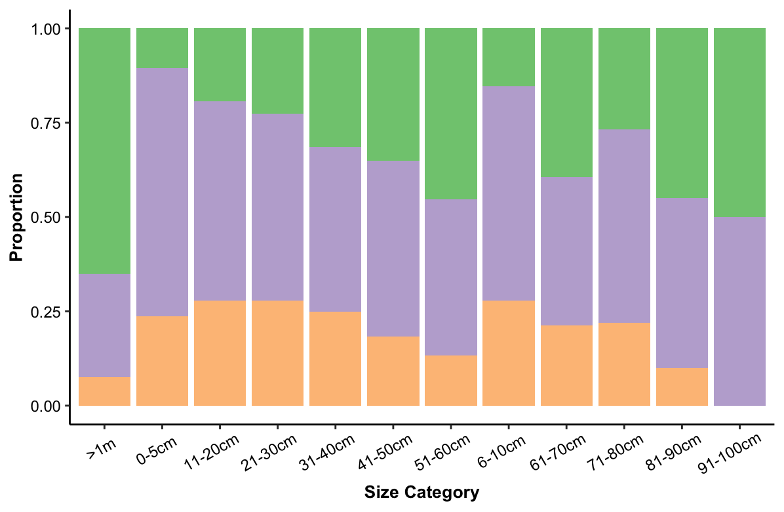


**Supplementary Figure 7.** Stacked bar chart showing proportion of each size category based on wave exposure levels.


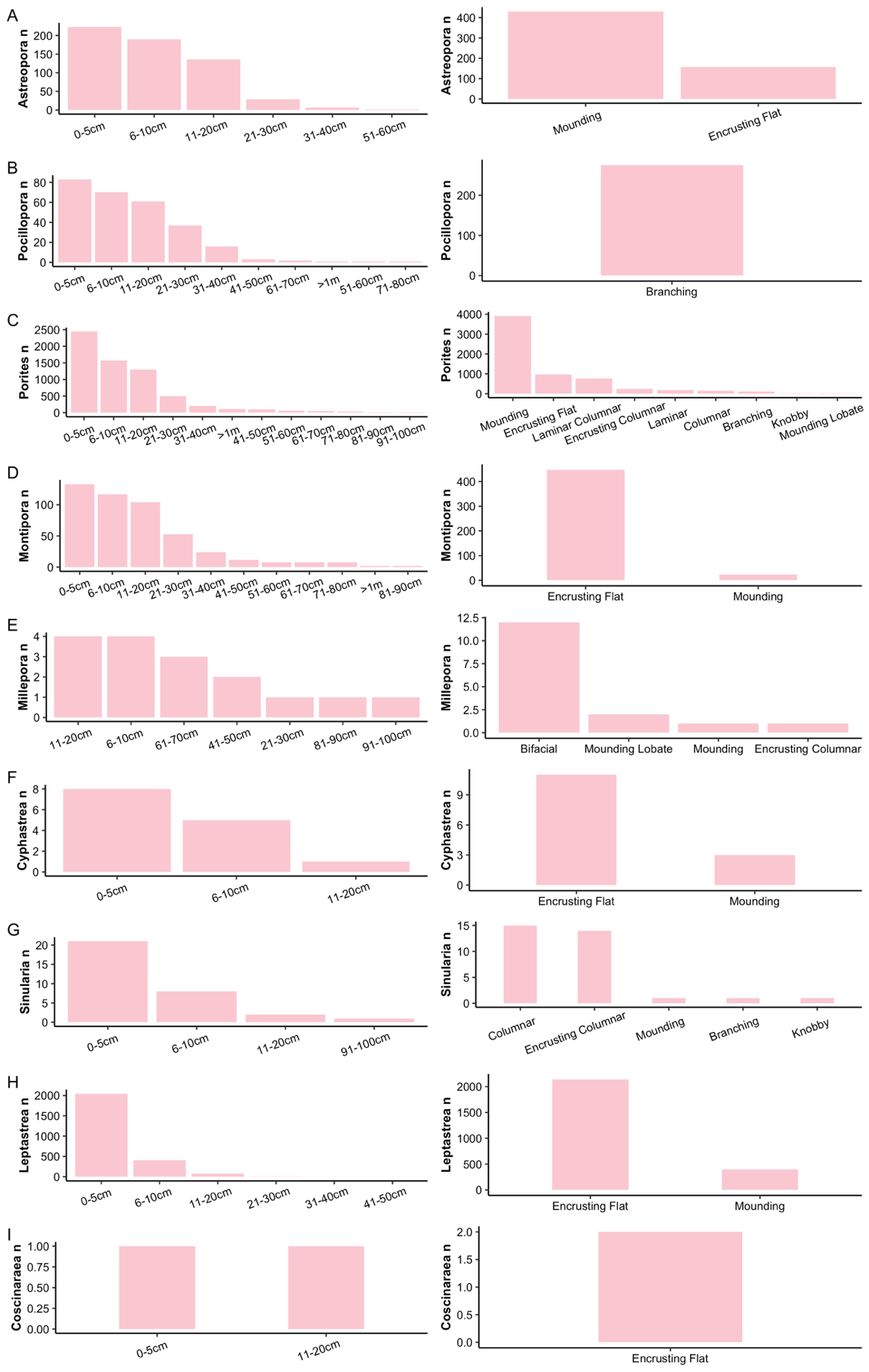


**Supplementary Figure 8.** Total proportion of each size category and morphology observed in (A) Astreopora, (B) Pocillopora, (C) Porites, (D) Montipora, (E) Millepora, (F) Cyphastrea, (G) Sinularia, (H) Leptastrea, and (I) Coscinaraea.


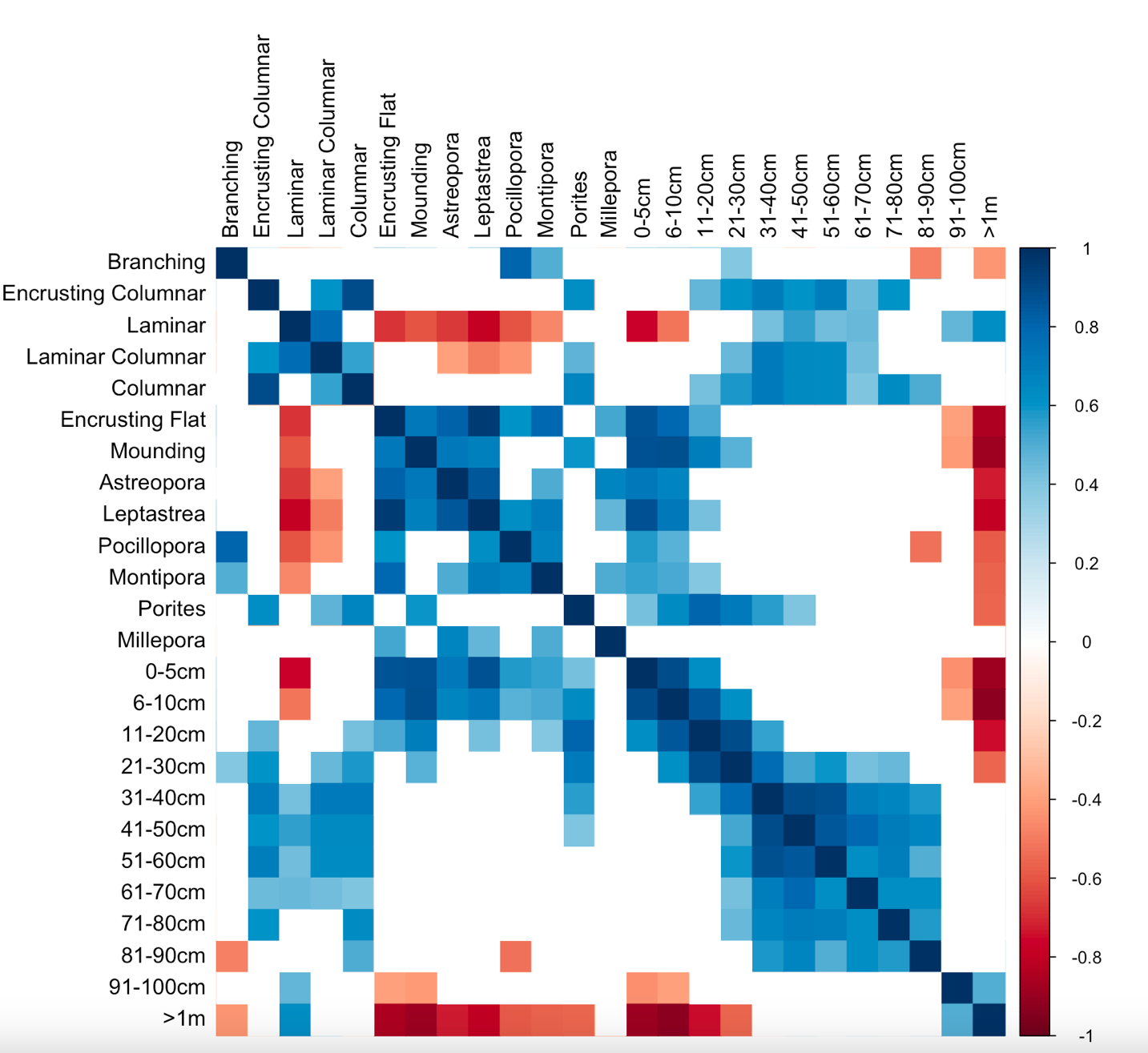


**Supplementary Figure 9.** Correlation matrix showing Spearman correlation values among all size categories and dominant morphologies and genera.
